# *Plasmodium vivax multidrug resistance*-*1* gene polymorphism in French Guiana

**DOI:** 10.1186/s12936-016-1595-9

**Published:** 2016-11-08

**Authors:** Emilie Faway, Lise Musset, Stéphane Pelleau, Béatrice Volney, Jessica Casteras, Valérie Caro, Didier Menard, Sébastien Briolant, Eric Legrand

**Affiliations:** 1 Laboratoire de Parasitologie, Institut Pasteur de la Guyane, Cayenne, French Guiana; 2 URPhyM-NARILIS, University of Namur, Namur, Belgium; 3 National Reference Center for Malaria, Institut Pasteur de la Guyane, Cayenne, French Guiana; 4 World Health Organization Collaborating Center for Surveillance of Antimalarial Drug Resistance, Institut Pasteur de la Guyane, Cayenne, French Guiana; 5 Environment and Infectious Risks unit, Genotyping of Pathogens Pole, Institut Pasteur, Paris, France; 6 Malaria Molecular Epidemiology Unit, Institut Pasteur in Cambodia, Phnom Penh, Cambodia; 7 Malaria Translational Research Unit, Institut Pasteur in Cambodia, Phnom Penh, Cambodia; 8 Direction Interarmées du Service de Santé, Cayenne, French Guiana; 9 Institut de Recherche Biomédicale des Armées, Brétigny sur Orge, France; 10 Malaria Translational Research Unit, Institut Pasteur, Paris, France; 11 Genetics and Genomics of Insect Vectors Unit, Institut Pasteur, Paris, France

## Abstract

**Background:**

*Plasmodium vivax* malaria is a major public health problem in French Guiana. Some cases of resistance to chloroquine, the first-line treatment used against *P. vivax* malaria, have been described in the Brazilian Amazon region. The aim of this study is to investigate a possible dispersion of chloroquine-resistant *P. vivax* isolates in French Guiana. The genotype, polymorphism and copy number variation, of the *P. vivax multidrug resistance gene*-*1* (*pvmdr1*) have been previously associated with modification of the susceptibility to chloroquine.

**Methods:**

The *pvmdr1* gene polymorphism was evaluated by sequencing and copy number variation was assessed by real-time PCR, in *P. vivax* isolates obtained from 591 symptomatic patients from 1997 to 2013.

**Results:**

The results reveal that 1.0% [95% CI 0.4–2.2] of French Guiana isolates carry the mutations Y976F and F1076L, and that the proportion of isolates with multiple copies of *pvmdr1* has significantly decreased over time, from 71.3% (OR = 6.2 [95% CI 62.9–78.7], p < 0.0001) in 1997–2004 to 12.8% (OR = 0.03 [95% CI 9.4–16.9], p < 0.0001) in 2009–2013. A statistically significant relationship was found between Guf-A (harboring the single mutation T958M) and Sal-1 (wild type) alleles and *pvmdr1* copy number.

**Conclusions:**

Few *P. vivax* isolates harboring chloroquine-resistant mutations in the *pvmdr1* gene are circulating in French Guiana. However, the decrease in the prevalence of isolates carrying multiple copies of *pvmdr1* might indicate that the *P. vivax* population in French Guiana is evolving towards a decreased susceptibility to chloroquine.

## Background

*Plasmodium vivax* remains the most geographically widespread of the five *Plasmodium* species infecting humans. As the second most common cause of malaria worldwide, *P. vivax* is the main cause of malaria in South America, where 390,000 cases were reported by the World Health Organization in 2015 [1]. Approximately 95% of these *P. vivax* malaria cases occur in nine countries of the Amazon Basin, namely Brazil, Bolivia, Colombia, Ecuador, French Guiana, Guyana, Peru, Suriname and Venezuela [1]. A total of 311 *P. vivax* cases were reported in French Guiana in 2014, representing 70% of the total number of malaria cases [2]. Since 1995, the treatments of uncomplicated *P. falciparum* malaria, mefloquine or halofantrine were used in monotherapy until 2002 when they were replaced by the association atovaquone–proguanil and in 2009 by the combination of artemether–lumefantrine [2–4]. Like the other countries across the continent, chloroquine is still recommended as the first-line treatment for *P. vivax* in French Guiana.

In 1989, the first cases of chloroquine-resistant *P. vivax* infection were reported in Papua New Guinea [5]. First cases of *P. vivax* resistance to chloroquine in South America were described in clinical studies of unsupervised chloroquine treatment in 1989 and 1992, in Colombia and Brazil, respectively [6, 7]. It was only in 1996 that the first confirmed clinical case of resistance was described in Brazil [8]. Chloroquine resistance has spread around the world over the last decade [9], and is now found in Southeast Asia [10–14] but also in Africa [15, 16], South America [8, 17–22] and the Middle East [23, 24].

Polymorphisms in *pvmdr1* gene (*P. vivax multidrug resistance*-*1* gene, PVX_080100), orthologous to *pfmdr1* gene in *Plasmodium falciparum* (PF3D7_0523000) [25], has been associated with chloroquine resistance in many studies. *Pvmdr1* Y976F and F1076L mutations are found in all malaria-endemic regions where chloroquine is used as the first-line treatment [13, 14, 26, 27]. Isolates bearing only F1076L mutations were identified but were not associated with a chloroquine resistance [28, 29]. This observation supports the argument that *P. vivax* chloroquine resistance requires the presence of both mutations. It has been suggested that F1076L is the prerequisite for the secondary acquisition of Y976F, which is responsible for the decrease in chloroquine susceptibility [29]. However, no other studies have observed this correlation between Y976F mutation and resistance phenotype [15, 18]. Several studies have pinpointed an increase in *pvmdr1* gene copy number, which seems to be related to an increased susceptibility to chloroquine [13, 14, 30, 31]. Furthermore, a recent study suggested that chloroquine resistance and clinical severity in vivax malaria were associated with increased expression levels of *pvmdr1* and *pvcrt*-*o* genes [32].

This study aims to estimate the possible emergence of chloroquine-resistant *P. vivax* in French Guiana. The work is divided into two parts. Firstly, *pvmdr1* gene polymorphism and copy number were assayed in *P. vivax* isolates obtained from blood samples of patients collected since 1997 and the temporal evolution of *pvmdr1* gene polymorphism and copy number were studied.

## Methods

### Sample collection

Between 1997 and 2013, samples were collected from 591 symptomatic patients presenting with malaria symptoms at health centres in French Guiana. *Plasmodium vivax* mono-infections were diagnosed by rapid diagnostic tests and/or microscopic examination of the blood. Blood samples were collected in EDTA-coated tubes and sent to the National Reference Centre (NRC) for Malaria at the *Institut Pasteur de la Guyane* for further analysis and biobanking.

### DNA extraction, amplification and sequencing

Parasite DNA was extracted from whole blood samples using the QIAamp DNA Blood Mini Kit (Qiagen, Courtaboeuf, France), following manufacturer’s instructions. The *pvmdr1* gene was amplified and sequencing using protocol (primers and amplification condition) previously described by Lekweiry et al. [33] except the polymerase, 0.025 U/μl of Ampli Taq Gold™, 2.5 mM MgCl_2_, 1× PCR Gold Buffer (Applied Biosystems). The amplification product was loaded on a 1.5% agarose gel and visualized after electrophoresis.

Sequencing of *pvmdr1* PCR product was performed by using the nested primers [33] generating a product length of 547 bp (region between codon 931 and 1095). These sequence were compared with the reference sequence Sal-1 (Genbank accession number AY571984).

### *Pvmdr1* and *pvaldolase* cloning

As a reference sample with a known *pvmdr1* copy number was not available, two reference plasmids containing one copy of the *pvmdr1* and *pvaldolase* genes were generated, respectively, to use as positive controls for the real-time PCR (qPCR). They were created by PCR amplification of *pvmdr1* and *pvaldolase* genes using A380 and A379 or A382 and A383 primers, respectively (Table 1). Each PCR product was cloned in pCR™4-TOPO^®^ plasmid using the TOPO^®^ TA Cloning^®^ kit (Invitrogen), following manufacturer’s protocol.

**Table 1:** Primers and probes for quantification of the *pvmdr1* copy number by qPCR

Primer and probe name	Sequence	Gene
A380	5′-GAG-AGG-ACG-TAA-ACG-TGC-TT-3′	*pvmdr1*
A379	5′-ACG-TTG-GTG-TCG-TAC-TGA-TTC-G-3′	
A399	5′-FAM_TTT-GCC-GCA-ATT-GA_MGB/NFQ-3′	
A382	5′-AGT-TTT-GTT-GGA-AGG-AGC-TTT-ATT-G-3′	*pvaldolase*
A383	5′-TGG-TTT-TCA-CAG-CAC-AGT-CGT-AT-3′	
A397	5′-FAM_CCC-AAC-ATG-GTG-ACC-G_MGB/NFQ-3′	

*MGB/NFQ* minor groove binder/non-fluorescent quencher, *pvmdr1 Plasmodium vivax* multi-drug resistance 1

### Real-time PCR to quantify the *pvmdr1* gene copy number

The *pvmdr1* gene copy number was measured by performing a qPCR in comparison to the reference gene *pvaldolase,* using the method previously described by Lekweiry et al. [33]. The reproducibility problems encountered were solved by designing new probes using Primer Express^®^ software (Applied Biosystems). These primers and probes are listed in Table 1. The qPCR was carried out in a 25 μl reaction volume containing 1 μl of DNA, 300 nM of each primer, 200 nM of probe, 12.5 μl of TaqMan^®^ Universal Master Mix II (Applied Biosystems) and water. Real-time PCR was performed under the following conditions: 95 °C for 10 min, then 40 cycles at 95 °C for 15 s and 65 °C for 1 min. Samples were set up in triplicate and experiments were repeated independently twice.

Results analysis was executed by StepOne™ software (Applied Biosystems). The signal from the *pvmdr1* gene was normalized to the single copy *pvaldolase* reference gene, then copy number was determined using the mathematical model described by Pfaffl [34].

### Statistical analysis

All statistical analyses were performed with R software (version 3.0.2). Percentages were calculated for each parameter studied, namely single nucleotide polymorphisms and increased *pvmdr1* copy number, in comparison to the total sample size. Corresponding 95% confidence intervals (CI) were calculated using the exact (Clopper-Pearson interval) method [35]. In this study, the *pvmdr1* copy number was analysed as a qualitative variable and two groups were considered: samples with one copy and samples with at least two copies of the *pvmdr1* gene.

A Chi square test for trend allowed comparison of the temporal evolution of *pvmdr1* allele frequencies and gene copy number. A logistic regression was used to determine the association between polymorphisms and the *pvmdr1* gene copy numbers. A p value below 0.05 was considered significant.

### Nucleotide sequence accession numbers

The Guf-A, Guf-B and Guf-C allele sequences of the *pvmdr1* gene reported in this study were deposited in GenBank under accession numbers KU196660, KU196661 and KU196662, respectively.

## Results

### Demographic information

A total of 547 patients for 591 sample, 362 men and 185 women with the sex ratio of 1.96, were included in this study. The average age was 29.2 years (1 month to 76 years, including 88 children under 15 years) with parasitaemia between 0.001 and 4% with an average of 0.32% (Table 2). Three time periods (1997–2004, 2005–2008 and 2009–2013) were considered according to the years 2005 when the *P. vivax* became the dominant species diagnosed in French Guiana, and 2009 when the combination of artemether and lumefantrine was adopted for the treatment of *P. falciparum* and mixed infections (*P. falciparum/P. vivax*) in French Guiana (Table 2). No significant association on all parameters (age, sex ratio and parasitaemia) and the time period.

**Table 2:** Demographic information

Year	Number of patient	Age (mini–max)	Nb men	Nb women	Sex ratio	Parasitemia % (mini–max)
1997–2004	136	26.7 (0.46–56)	85	50	1.7	0.4 (0.01–2)
2005–2008	120	22.9 (0.08–63)	82	38	2.16	0.5 (0.01–4)
2009–2013	291	31.2 (0.67–76)	195	97	2.01	0.3 (0.001–2.3)
Total	547	29.2 (0.08–76)	362	185	1.96	0.32 (0.001–4)

Age were indicated in year; the parasitemia were indicated on percentage of red blood cell infected by *P. vivax*

### Polymorphism of the *pvmdr1* gene

**Fig. 1:**
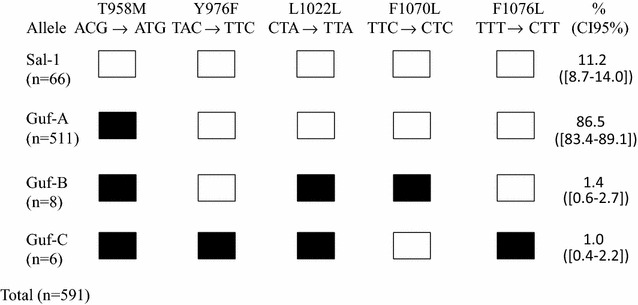
*Pvmdr1* sequence polymorphism (codon 931–1095) of 591 isolates collected in French Guiana between 1997 and 2013. Only polymorphic codons are indicated. *Open symbols* denote the wild-type (Sal-1-type) nucleotide sequence, and *black symbols* indicate the presence of the mutant sequence shown at the *top*. The number of isolates observed for each allele is indicated in *brackets*

Among the 591 genotyped samples, four non-synonymous mutations (T958 M, Y976F, F1070L and F1076L) and one synonymous mutation (L1022L) were identified. The French Guiana strains were then divided into four alleles (Fig. 1). The Sal-1 wild-type allele was present in 11.2% (n = 66/591, CI 95% [8.7–14.0]) of the samples while 86.5% (n = 511/591, CI 95% [83.4–89.1]) of isolates carried the T958 M mutation and this predominant allele was called Guf-A. Only 1.4% (n = 8/591, CI 95% [0.6–2.7]) of isolates carried the T958M/F1070L mutations and this double mutant allele was referenced as Guf-B. Finally, 1.0% (n = 6/591, CI 95% [0.4–2.2]) of isolates carried the T958M/Y976F/F1076L, this triple mutant was named Guf-C (Fig. 1).

**Fig. 2:**
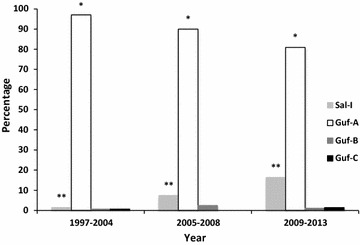
Temporal evolution of *pvmdr1* allele frequencies. *p < 0.0002 and **p < 0.0001 denote significant differences in allele frequencies through time (Chi square test for trend)

The temporal evolution of these alleles was then determined (1997–2004, n = 136; 2005–2008, n = 120 and 2009–2013, n = 335; Fig. 2). No significant association was found between the frequency of Guf-B and Guf-C alleles and the time period (p = 0.44 and p = 0.35, respectively, Chi square test for trend). A statistically significant increase in the frequency of the Sal-1 allele through time (p < 0.0001) has detected, along with a statistically significant decrease for the Guf-A allele (p < 0.002) (Fig. 2). Furthermore, relationship between epidemiological data and genotype was not observed.

### Relationship between copy number and genotype of the *pvmdr1* gene

**Fig. 3:**
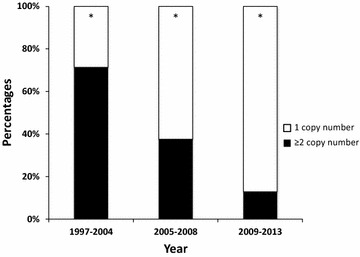
Temporal evolution of the proportion of isolates harbouring single or multiple *pvmdr1* copy number. *White* represents the isolates with single copy number of *pvmdr1* gene, and *black* indicates isolates with two or more *pvmdr1* copy numbers. *p < 0.0001 for Chi square test for trend

The copy number of the *pvmdr1* gene was determined for all samples and varied from 1 to 8 (mean 1.45, CI 95% [1.35–1.55]). The majority of the sample, 68.5% (n = 405/591, CI 95% [64.6–72.3]), had one copy while 31.5% (n = 186/591, CI 95% [27.7–35.4]) had two to eight copies. The frequency of isolates with multiple copies of the *pvmdr1* gene was significantly higher in samples collected between 1997 and 2004 (97/136, 71.3%, CI 95% [62.9–78.7]) than in samples from 2005 to 2008 (45/120, 37.5%, CI 95% [28.8–46.8]) or even after 2008 (43/335, 12.8%, CI 95% [9.4–16.9]). This decrease over time was statistically significant (p < 0.0001, Chi square test for trend, see Fig. 3). Moreover, relationship between epidemiological data and copy number was not observed.

**Fig. 4:**
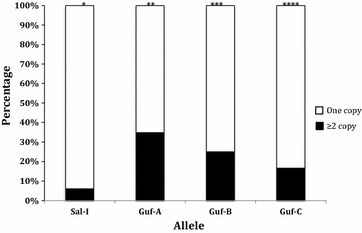
Evaluation of the relationship between *pvmdr1* copy number and genotype. *OR = 0.12 [95% CI 0.04–0.034], p = 0.00006. **OR = 5.57 [95% CI 2.51–12.36], p = 0.00002. ***OR = 0.73 [95% CI 0.15–3.64], p = 0.70. ****OR = 0.44 [95% CI 0.05–3.76], p = 0.45

The association between the copy number and genotype of the *pvmdr1* gene was evaluated. The proportion of isolates harbouring multiple copies was not equally distributed among the different alleles. A statistically significant association was found between the Sal-1 allele and single copy *pvmdr1* gene (p = 0.0006) as well as between the Guf-A allele and copy number greater than 1 (p = 0.0002). No statistically significant relationship was found between the Guf-B and Guf-C alleles and the *pvmdr1* gene copy number (p = 0.93 and p = 0.58, respectively, Fig. 4).

## Discussion

Vivax malaria is a major public health problem in South America. In French Guiana, it is currently responsible for two-thirds of malaria cases. Resistance to chloroquine, the main treatment used against vivax malaria [2], has been reported in the Brazilian Amazon Region [4, 17, 18, 20, 21, 32, 36, 37]. In French Guiana, the development of gold-mining activities and the consequent human migration between French Guiana and neighbouring countries, Brazil and Suriname [4, 38, 39], have raised fears that chloroquine-resistant *P. vivax* isolates may spread. It is therefore important to follow the circulation of resistant isolates. There are previous studies suggesting that Y976F and F1076L mutations in the *pvmdr1* gene are associated with in vitro resistance to chloroquine [13, 14]. *Pvmdr1* Y976F mutation alone is not sufficient to cause the failure of chloroquine treatment, as observed in Madagascar [15], Brazil [21] and Honduras [40]. It can only affect treatment outcome when associated with the F1076L mutation. In French Guiana, these two mutations were carried by 1.0% of the isolates (i.e., Guf-C allele). Therefore, these parasites could potentially be resistant to chloroquine; however, an association with clinical drug response or in vitro susceptibility have not been investigated. This value is similar to the 1.8% prevalence reported in Brazil [41].

Sal-1 and Guf-A alleles were present in 11.2 and 86.5% of the samples in French Guiana, respectively, showing significant and inverse trends in the temporal evolution of their frequency; while the frequency of the Sal-1 allele significantly increased over time, the frequency of the Guf-A allele decreased. These temporal variations of allele frequencies could be explained by different factors, such as the increased circulation of isolates between French Guiana and Brazil or changes in drug policy for the treatment of *P. falciparum*. This is supported by a recent study showing that the T958M mutation allele is the majority among Brazilian isolates collected between 2010 and 2014 [42]. Many studies in Southeast Asia have shown that isolates with *pvmdr1* gene amplification were characterized by increased susceptibility to chloroquine but decreased susceptibility to mefloquine [13, 31]. A significant decrease of the proportion of isolates with multiple copies of *pvmdr1* over this 16-year study period has been found, decreasing from 71.3% between 1997 and 2004 to only 12.8% between 2009 and 2013. A recent study comparing *P. vivax* isolates from French Guiana (collected between 2001 and 2003) and Southeast Asia (collected in 2010) showed the number of isolates with multiple copies of *pvmdr1* gene to be higher in French Guiana than in Cambodia [43]. Moreover, *pvmdr1* gene amplifications were rare (fewer than 2%) in countries where mefloquine has never been used for malaria treatment, such as Madagascar and Sudan [43]. In *P. falciparum*, multiple copies of *pfmdr1* were associated with mefloquine-resistant isolates [44]. This has been confirmed in French Guiana where isolates with amplified copy number of *pfmdr1* gene were significantly correlated with resistance to mefloquine and halofantrine, both used in monotherapy against uncomplicated *P. falciparum* malaria until 2002 [45]. *P. vivax* isolates were therefore subjected to indirect selection pressure by mefloquine during the treatment of *P. falciparum* or mixed infections (*P. falciparum* and *P. vivax*). When the use of mefloquine and halofantrine ceased in French Guiana, *P. falciparum* isolates with one copy of *pfmdr1* increased [45]. This loss of selective pressure would also explain the increased frequency of *P. vivax* isolates with a single copy of the *pvmdr1* gene, a genotype associated to chloroquine resistance [13].

Recently two studies analyzing the whole genome sequences of isolates collected in America, Africa and Asia, have shown great diversity of *P. vivax* isolates according to their geographical origin in particular for malaria drug antifolate resistance genes involved in resistance to (*pvdhfr* and *pvdhps*) [46, 47]. Nevertheless, the role of *pvmdr1* in conferring resistance to chloroquine is still elusive and controversial and was recently further challenged by global population genomic studies of *P. vivax*. Indeed, Schousboe et al. studied the prevalence of polymorphisms and the diversity in microsatellite markers flanking the *pvmdr1* gene in *P. vivax* isolates from seven endemic countries worldwide (Pakistan, Afghanistan, Nepal, Sri Lanka, Ecuador, Sao Tomé and Sudan). Although they showed that Y976F and F1076L mutations in *pvmdr1* gene have developed on multiple haplotype backgrounds by convergent evolution in these countries, they highlighted high levels of diversity around mutant alleles, suggesting these alleles were not subject to a selective sweep [48].

## Conclusions

The present study indicates that *P. vivax* isolates with mutations in *pvmdr1* previously described as associated with chloroquine resistance are present at low frequency in French Guiana. In addition, the number of copies of the gene decreases over time. A continuous surveillance of these genetic markers in the *P. vivax* population circulating in this region should be maintained to ensure public health.
